# Knowledge, attitudes, practices and intention to get vaccinated against COVID-19: results from a cross-sectional survey in three peri-urban communities in South Africa

**DOI:** 10.11604/pamj.2023.45.120.37210

**Published:** 2023-07-12

**Authors:** Renay Weiner, Sarah Magni, Tetelo Maakamadi, Tamika Fellows, Susan Aitken, Jessica Yun, Stefano Tempia, Anne von Gottberg, Jinal Bhiman, Sibongile Walaza, Jocelyn Moyes, Cherie Cawood, Neil Martinson, Limakatso Lebina, Cheryl Cohen, Nicole Wolter

**Affiliations:** 1 Genesis Analytics, Johannesburg, South Africa,; 2 Faculty of Health Sciences, School of Public Health, University of the Witwatersrand, Johannesburg, South Africa,; 3 Research and Training for Health and Development, Johannesburg, South Africa,; 4 Centre for Respiratory Diseases and Meningitis, National Institute for Communicable Diseases (NICD) of the National Health Laboratory Service, Johannesburg, South Africa,; 5 Faculty of Health Sciences, School of Pathology, University of the Witwatersrand, Johannesburg, South Africa,; 6 Epicentre Health Research, Durban, South Africa,; 7 Perinatal HIV Research Unit (PHRU), University of the Witwatersrand, Johannesburg, South Africa,; 8 Johns Hopkins University Center for Tuberculosis Research, Baltimore, Maryland, United States of America

**Keywords:** COVID-19, health knowledge, attitudes, practice, South Africa

## Abstract

**Introduction:**

South Africa has the largest number of confirmed cases of COVID-19 in Africa. Data to inform public health strategies to mitigate the spread of new variants and severity of disease is needed, including information on knowledge, attitudes and practices (KAP) regarding COVID-19, factors associated with intention to get vaccinated, and viewpoints on reliable sources of data.

**Methods:**

we investigated these topics as part of the COVID-19 healthcare utilization and seroprevalence (HUTS) cross-sectional survey in three communities in South Africa: Mitchell´s Plain (Western Cape Province), Pietermaritzburg (KwaZulu-Natal Province) and Klerksdorp (North West Province) during and after the second wave of COVID-19 prior to vaccine availability.

**Results:**

primary caregivers from 5799 households participated in the study, 41.1% from Pietermaritzburg, 34.2% from Klerksdorp and 24.7% from Mitchells Plain. Two-thirds and 94.7% of respondents had correct knowledge on the cause and spread of COVID-19, respectively. Knowledge measures were significantly associated with age less than 65 years, the highest level of education and site (Mitchells Plain). Desired preventive behaviors were associated with higher socio-economic status. While 64.7% of people intended to get vaccinated, those over 64 years of age were more likely to intend to vaccinate (aOR: 1.25, 95% CI: 1.06-1.47). Vaccine intention related to protection of self (58.4%) and family (40.0%). The most trusted source of COVID-19 information was television (59.3%) followed by radio (20.0%).

**Conclusion:**

these data can be used to design targeted public health campaigns for the current COVID-19 and future epidemics, ensuring that socio-economic constraints and preference for trusted information are considered.

## Introduction

**Figure 1:**
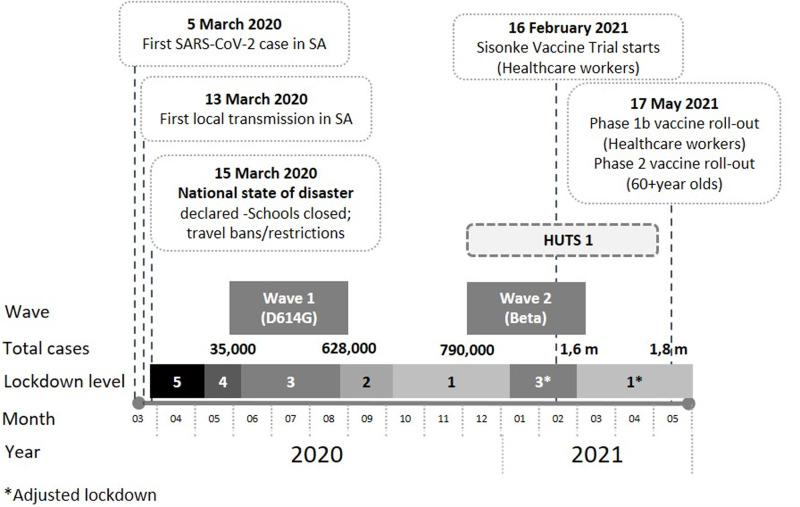
timeline of COVID-19 infections and vaccination roll-out in relation to the healthcare utilisation and seroprevalence study in South Africa

South Africa has been severely affected by the novel coronavirus disease (COVID-19) epidemic despite the early implementation of a lockdown, promotion of non-pharmaceutical interventions (NPI) and more recently vaccine roll-out. By August 2022, over 4 million cases had been laboratory confirmed and over 100 000 people had died due to the disease [1], with the excess deaths estimated to be over 320 000 [2]. Prevention is key to the public health response to the pandemic and includes both behaviour change and vaccination. The World Health Organization advises that the public maintains physical distance, mask wearing and handwashing as non-pharmaceutical interventions (NPI) to avoid spreading and contracting COVID-19 [3]. Similarly, these behaviors have been promoted by the South African National Department of Health and mask wearing became mandatory in public as part of the amendment to the Disaster Management Act during the earlier waves of the pandemic [4]. Despite the centrality of these preventive measures, peer-reviewed data are limited, particularly from community-based studies on knowledge, attitudes and practices (KAP) including intention to vaccinate for COVID-19 prevention in South Africa. In addition to NPI, vaccinating South Africans is an essential step towards reducing both the severity and mortality from COVID-19 infections. Data demonstrates that the Pfizer-BioNTech vaccine, the most widely used vaccine in South Africa, is effective and leads to reductions of 80%-85% in COVID-19 hospitalizations associated with the Delta variant and a 70% decrease in Intensive care unit (ICU) or high care admission associated with the Omicron variant [5]. Large-scale uptake of the COVID-19 vaccine is required to ensure that as many people as possible are reached and to curb the emergence of new variants for which the effectiveness of current vaccines may be lower. Success of prevention programs are reliant on individual behaviour change which are in turn informed by knowledge and attitudes, including self-efficacy. Information on KAP related to prevention through both NPI and vaccination will assist with strengthening the COVID-19 prevention response including social and behaviour change communication strategies. Moreover, data generated will inform future pandemic preparedness enabling a faster and more targeted approach to behavioral interventions. The aim of the study was to assess KAP related to COVID-19 prevention from a household survey in three South African communities. The study was conducted three months prior to the introduction of the vaccine and hence it was not possible to investigate vaccination uptake (Figure 1). Specifically, the objectives were to: i) Describe and identify factors associated with KAP in relation to COVID-19 prevention in three South African sites; ii) assess predictors of intention to be vaccinated to prevent COVID-19 and related reasons and; iii) identify trusted sources of COVID-19 information.

## Methods

**Design:** this study was part of a larger survey of COVID-19 healthcare utilization and seroprevalence (HUTS) conducted in three peri-urban communities in South Africa, namely Mitchell´s Plain (Western Cape Province), Pietermaritzburg (KwaZulu-Natal Province) and Klerksdorp (North West Province) [6]. A cross-sectional survey of randomly selected households using global positioning system co-ordinates (GPS) was conducted between November 2020 and April 2021 which was during and after the second wave of the COVID-19 epidemic in South Africa.

**Sampling:** the sample size was based on primary outcomes of the HUTS study using a one stage cluster sampling design [6]. Of the 7032 households that were available when visited by the field work team, 5813 primary caregivers were at home and willing to participate (17.3% refused). Those that gave informed consent and who were 18 years or older were included in the study, totaling 5799 primary caregivers. The primary caregiver of each selected household was defined as someone identified by the people in the household as the person who is most involved in the daily care of the household members.

**Data collection:** trained fieldworkers administered structured questionnaires electronically in the home language of the primary caregiver using Research Electronic Data Capture (REDCap, Vanderbilt University, USA). Data were collected on household socio-economic status, demographic characteristics, impact of COVID-19 lockdown on household income, knowledge, attitudes and practices related to COVID-19 prevention including intention to be vaccinated.

**Measurement:** sociodemographic variables included: sex, age, level of completed education for all household members, type of dwelling, socio-economic status (SES) measured by asking the primary caregiver “Which of the following items does your household have in the house?” with 28 possible items such as hot running water, electricity and television ownership which were added and allocated to low, medium and high socioeconomic groups, impact of COVID-19 lockdown on household income and site (Mitchell´s Plain, Pietermaritzburg, and Klerksdorp). Knowledge of the cause of COVID-19 and transmission were deemed correct if the individual responded that COVID-19 is caused by the response option “virus/bacteria/germs” and spreads either by “coughing and sneezing, talking, handshakes or hugs, touching an item someone else touched”. In line with other studies [7], intention to get vaccinated was measured by asking respondents: “If a COVID-19 vaccine were to become available, how likely would you be to choose to get the vaccine for yourself?”. Response options were “very likely, somewhat likely, not too likely and not at all likely” and a variable which combined “very likely” and “somewhat likely” was used to measure overall vaccination intention. Respondents were also asked to provide reasons as to why they did or did not intend to get vaccinated with the following open-ended questions ”What are reasons that you would choose to get the COVID-19 vaccine“ and ”What are reasons that you would choose not get the COVID-19 vaccine?“ To ascertain information sources, respondents were asked what source of COVID-19 related information they trusted the most. A list of 14 options were read out and the respondent was asked to select one, which included an ”other“ option.

**Analysis:** descriptive analysis was undertaken to summarize the distribution of demographic characteristics of individuals by study site and percentages of most trusted sources of information were calculated. Measures of knowledge, attitudes and practices pertaining to mask wearing, handwashing, social distancing, self-efficacy were assessed by sociodemographic characteristics, and chi-squared tests applied to assess significance. To assess the predictors of intention to get vaccinated, a random effects logistic regression analysis was conducted, adjusting for clustering by study site. A multivariable model was built using backward stepwise selection, starting with variables that were significant at p<0.2 at univariate analysis. Variables with missing data were coded as missing and this was retained as a category in the multivariable model. This was done to minimize bias because for some sensitive questions e.g. level of education or impact of the lockdown on household income responses were incomplete, but we felt non-responders could differ from responders. The results were reported using adjusted Odds Ratios (aORs) and 95% confidence intervals (CIs). Statistical significance was assessed at p<0.05. Stata 17 was used to conduct all statistical analysis (StataCorp Limited, College Station, Texas, USA).

## Results

Of the 5799 primary caregivers who participated in the study, 2381 (41.1%) were from Pietermaritzburg, 1983 (34.2%) from Klerksdorp and 1435 (24.7%) from Mitchells Plain. More than two-thirds (68.1%, n= 3951) of respondents were female, and this pattern was consistent across the sites. Mitchells Plain had a higher proportion of respondents with no or only primary school education (27.5% versus 16.8% and 22.7% for Pietermaritzburg and Klerksdorp respectively) and lower proportion in the 18-49 year age group (43.8%) compared to the other two sites (59.6% and 61.3% for Pietermaritzburg and Klerksdorp respectively). Table 1 presents knowledge and self-efficacy related to COVID-19 prevention by socio-demographic status. Two-thirds (66.2%, n=3840) and 94.7% (n=5489) of respondents had correct knowledge on the cause and spread of COVID-19 respectively. Both these knowledge measures increased significantly in specific age bands (50-64 years and 18-49 years respectively), with higher level of education completed, by site (Mitchells Plain), and with increased household income during lockdown regulations. Confidence to take action (self-efficacy) to prevent getting COVID-19 was 65.7% (n=3808) overall. Self-efficacy increased with some level of secondary education completed, higher socio-economic status, site (Klerksdorp), residing in a house/flat as well as decreased or no impact on household income during lockdown regulations. (p≤0.05). While both knowledge measures were associated with increasing education levels, this was not the case for self-efficacy which was decreased for those with tertiary education compared to those with some secondary education. Table 2 presents behavioural practices to prevent spread of COVID-19. Mask wearing was prevalent across the sites (95.6%, n=5543) and was more commonly practiced than frequent handwashing (72.1% n= 4178) or social distancing (77.9% n=4520). All desired behavioral practices were significantly associated with medium or high socio-economic status, whilst those in the low socio-economic category were consistently less likely to practice desired behaviours. Using soap/sanitizer was more common amongst females compared to males (p=0.001) while frequent handwashing was linked to increasing age (p=0.032). Consistently, a lower proportion of respondents from the Pietermaritzburg site practiced preventive behaviour. All preventive behaviours were most commonly practiced amongst participants who lived in a house/flat and least commonly practiced by those who resided in traditional housing such as a mud hut.

**Table 1:** knowledge and self-efficacy related to COVID-19 prevention by socio-demographic status, N=5799

	Correct knowledge on cause of COVID-19			Correct knowledge on spread of COVID-19			Confidence to take action to prevent getting COVID-19		
	N	%	p-value	N	%	p-value	N	%	p-value
	3840	66.2		5489	94.7		3808	65.7	
**Sociodemographic characteristics**									
**Sex**									
Male	1256	68.0	0.054	1768	95.7	0.019*	1199	64.9	0.389
Female	2584	65.4		3721	94.2		2609	66.0	
**Age in years**									
18-49	2115	64.9	0.008*	3112	95.5	0.003*	2127	65.3	0.392
50-64	1173	69.2		1594	94.0		1135	67.0	
>64	552	65.4		783	92.8		546	64.7	
**Highest level of education completed**									
None/primary schooling	762	61.2	<0.001**	1143	91.8	<0.001**	808	64.9	0.001*
Some secondary	1403	66.0		2014	94.7		1456	68.5	
Grade 12	1280	69.1		1780	96.1		1199	64.7	
Tertiary	371	68.3		523	96.3		323	59.5	
No response/not sure	24	75.0		29	90.6		22	68.8	
**Socioeconomic status**									
Low	1433	72.8	<0.001**	1;874	95.2	0.426	1242	63.1	0.003*
Medium	1263	63.7		1873	94.5		1302	65.7	
High	1144	61.9		1742	94.3		1264	68.4	
**Site**									
Pietermaritzburg	1523	64.0	<0.001**	2243	94.2	<0.001**	1262	53.0	<0.001**
Klerksdorp	1165	58.8		1850	93.3		1546	78.0	
Mitchells Plain	1152	80.3		1396	97.3		1000	69.8	
**Type of dwelling**									
House/flat	3467	66.7	0.009*	4924	94.7	0.781	3472	66.7	<0.001**
Traditional house (e.g. mud hut)	104	57.5		170	93.9		73	40.3	
Informal house (shack)	263	65.3		383	95.0		255	63.3	
Other	1	16.7		6	100.0		5	83.3	
No response/not sure	5	71.4		6	85.7		3	42.9	
**Impact of lockdown on household income**									
No impact	2500	68.1	<0.001**	3497	95.2	0.013*	2398	65.3	0.014*
Gained more money	98	77.8		123	97.6		72	57.1	
Lost money	1198	61.9		1808	93.4		1303	67.3	
No response/not sure	44	67.7		61	93.9		35	53.9	

* p-value <0.05 ; **p-value<0.001

**Table 2:** behavioural practices to prevent COVID-19 spread by socio-demographic characteristics, N=5799

	More frequent handwashing since COVID-19 wave 1 or 2 lockdowns		Always use soap/sanitiser when hand washing		Wear a face mask due to COVID-19		Almost always wear a face mask when using public transport		Keep 2m away from people not in household (often/always)	
	n (%) = 4148 (72.1)		n (%) = 3773 (65.1)		n (%) = 5543 (95.6)		n (%) = 5341 (92.1)		n (%) = 4520 (77.9)	
**Demographic**	**n (%)**	**p-value**	**n (%)**	**p-value**	**n (%)**	**p-value**	**n (%)**	**p-value**	**n (%)**	**p-value**
**Sex**										
Male	1314 (71.1)	0.274	1148 (62.1)	0.001*	1773 (95.9)	0.367	1675 (90.6)	0.005*	1417 (76.7)	0.112
Female	2864 (72.5)		2625 (66.4)		3770 (95.4)		3666 (92.8)		3103 (78.5)	
**Age in years**										
18-49 years	2306 (70.7)	0.032*	2090 (64.1)	0.215	3113 (95.5)	0.260	2990 (91.7)	0.156	2479 (76.0)	<0.001**
50-64 years	1242 (73.3)		1120 (66.1)		1630 (96.2)		1579 (93.2)		1355 (79.9)	
>64 years	630 (74.6)		563 (66.7)		800 (94.8)		772 (91.5)		686 (81.3)	
**Highest level of education completed**										
None/primary schooling	876 (70.4)	0.082	800 (64.3)	0.414	1185 (95.2)	0.219	1110 (89.2)	<0.001**	932 (74.9)	0.027*
Some secondary	1543 (72.5)		1366 (64.3)		2036 (95.7)		1984 (93.3)		1692 (79.6)	
Grade 12	1346 (72.7)		1219 (65.8)		1781 (96.2)		1717 (92.7)		1 439 (77.7)	
Tertiary	396 (72.9)		369 (68.0)		510 (93.9)		499 (91.9)		432 (79.6)	
No response/not sure	17 (53.1)		19 (59.4)		31 (96.9)		31 (96.9)		25 (78.1)	
**Socioeconomic**										
Low	1370 (69.6)	0.001*	1119 (56.8)	<0.001**	1860 (94.5)	0.004*	1773 (90.1)	<0.001**	1467 (74.5)	<0.001**
Medium	1484 (74.9)		1307 (65.9)		1915 (96.6)		1 847 (93.2)		1523 (76.8)	
High	1324 (71.7)		1347 (72.9)		1768 (95.7)		1721 (93.1)		1530 (82.8)	
**Site**										
Pietermaritzburg	1469 (61.7)	<0.001**	1253 (52.6)	<0.001**	2 2167 (91.0)	<0.001**	2070 (86.9)	<0.001**	1634 (68.6)	<0.001**
Klerksdorp	1545 (77.9)		1475 (74.4)		1 955 (98.6)		1918 (96.7)		1629 (82.2)	
Mitchells Plain	1164 (81.1)		1045 (72.8)		1421 (99.02)		1353 (94.3)		1257 (87.6)	
**Impact of lockdown on household income**										
No impact	2577 (70.2)	<0.001**	2321 (63.2)	<0.001**	3454 (94.1)	<0.001**	3336 (90.9)	<0.001**	2864 (78.0)	0.860
Gained more money	96 (76.2)		85 (67.5)		119 (94.4)		114 (90.5)		95 (75.4)	
Lost money	1464 (75.6)		1329 (68.7)		1908 (98.6)		1831 (94.6)		1512 (78.1)	
No response/not sure	41 (63.1)		38 (58.5)		62 (95.4)		60 (92.3)		49 (75.4)	
**Type of dwelling**										
House/Flat	3796 (73.0)	<0.001**	3481 (66.9)	<0.001**	4999 (96.1)	<0.001**	4850 (93.2)	<0.001**	4122 (79.2)	<0.001**
Traditional house (e.g. mud hut)	92 (50.8)		65 (35.9)		161 (89.0)		124 (68.5)		95 (52.5)	
Informal house (shack)	278 (69.0)		218 (54.1)		371 (92.1)		355 (88.1)		292 (72.5)	
Other	6 (100)		5 (83.3)		6 (100)		6 (100)		6 (100)	
No response/not sure	6 (85.7)		4 (57.1)		6 (85.7)		6 (85.7)		5 (71.4)	

* p-value <0.05; **p-value < 0.001

**Figure 2:**
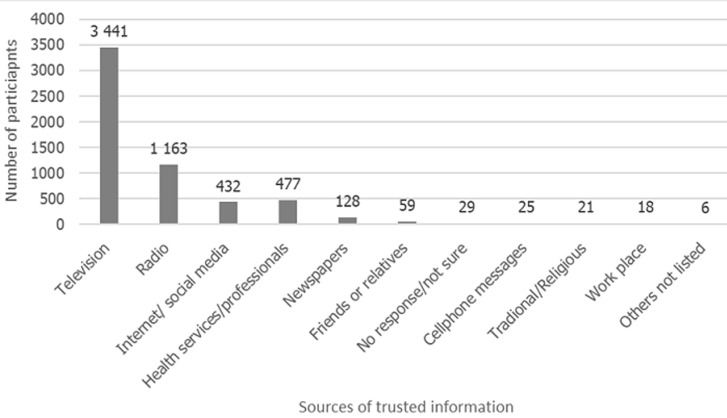
most trusted source of information for receiving information about COVID-19 N=5799

Table 3 presents the proportions of those who intended to get vaccinated, as well as predictors thereof. While 64.7% (n=3752) of respondents intended to get vaccinated, those over the age of 64 years were more likely to intend to vaccinate (adjusted odds' ratio (aOR): 1.25, 95% confidence interval (CI): 1.06-1.47). Losing household income as a result of the lockdown regulations was significantly associated with being less likely to intend to get vaccinated (aOR 0.75, 95% CI: 0.72 - 0.78). Wearing a face mask was significantly associated with intention to vaccinate on univariate analysis, and this association remained statistically significant in the adjusted analysis (aOR 1.53, 95% CI: 1.07-2.21). Most common reasons given for intending to vaccinate were to protect oneself from getting COVID-19 (58.4% n=3386) and protect other members of the family (40.0% n=2320). Worrying about the safety of the vaccine (14.1% n=820), not believing that the vaccine works (12.0% n=694) and thinking that the vaccine might be expensive (4.1% n=237) were the most commonly cited reasons for not seeking to vaccinate. The data indicate that the most trusted source of COVID-19 information was television (59.3%; n=3441) followed by radio (20.0%; n=1163), health services/professionals (8.2%; n=477), and internet/social media (7.4%; n=432). Remaining sources accounted for less than 5.0% (n=286), and comprised of newspapers, friends/relatives, phone messages, workplace, and traditional/religious leaders (Figure 2).

**Table 3:** crude and adjusted odds ratios* for predictors of intention to get vaccinated N=5799

Predictor	n	%	Univariate analysis OR (95% CI)*	P-value	Multivariable analysis aOR (95% CI)	P-value
**Sex**						
Male	1224	32.6	Ref		Ref	
Female	2528	37.4	0.91 (0.74 – 1.12)	0.351	0.902 (0.77 – 1.06)	0.218
**Age in years**						
18-49	2076	55.3	Ref		Ref	
50-64	1098	29.3	1.05 (0.77 – 1.43)	0.764	1.05 (0.82 – 1.35)	0.674
>64	578	15.4	1.24 (0.98 – 1.57)	0.079	1.25 (1.06 – 1.47)	0.007**
**Level of education**						
None/primary schooling	809	21.6	Ref		Ref	
Some secondary	1390	37.0	1.02 (0.64 – 1.62)	0.945	1.08 (0.69 – 1.68)	0.735
Grade 12	1207	32.2	1.01 (0.66 – 1.55)	0.969	1.12 (0.80 – 1.59)	0.502
Tertiary	331	8.8	0.84 (0.57 – 1.24)	0.387	0.97(0.70-1.59)	0.873
No response/not sure	15	0.4	0.48 (0.29-0.77)	0.002**	0.49 (0.31 – 0.78)	0.003**
**Socio-economic status**						
Low	1294	34.5	Ref		Ref	
Medium	1317	35.1	1.03 (0.817 – 1.307)	0.786	1.03 (0.82 – 1.29)	0.823
High	1141	30.4	0.84 (0.662 – 1.071)	0.160	0.85 (0.67 – 1.07)	0.163
**Impact of lockdown on household income**						
No impact	2465	65.7	Ref		Ref	
Gained more money	79	2.1	0.82 (0.605 – 1.119)	0.214	0.84 (0.58 – 1.21)	0.348
Lost money	1176	31.3	0.76 (0.709 – 0.810)	<0.001***	0.75 (0.72 – 0.78)	<0.001***
No response/not sure	32	0.9	0.47 (0.378 – 0.596)	<0.001***	0.49 (0.36-0.66)	<0.001***
**More frequent handwashing since COVID-19**	;					
No	959	25.6	Ref			
Yes	2793	74.4	1.39 (0.72 – 2.70)	0.328		
**Always use soap/sanitiser when hand washing**						
No	1271	33.9	Ref			
Yes	2 481	66.1	1.14 (0.79 – 1.64)	0.477		
**Wear a face mask due to COVID-19**						
No	139	3.7	Ref	;	Ref	
Yes	3613	96.3	1.58 (1.27 – 1.95)	<0.001***	1.53 (1.07 – 2.21)	0.020**
**Almost always wear a face mask when using public transport**						
No	264	7.0	Ref			
Yes	3488	93	1.38 (0.94 – 2.04)	0.101		
**Confident to take action to prevent getting COVID-19**						
No	1197	31.9	Ref			
Yes	2;555	68.1	1.35 (0.85 – 2.16)	0.204		

* ORs were adjusted for clustering; **p-value <0.05 ; ***p-value<0.001

## Discussion

This study of knowledge, attitudes and practices related to COVID-19 prevention amongst caregivers in three communities in South Africa found very high knowledge levels on the spread of COVID-19, moderately high knowledge levels on the cause of COVID-19 and moderately high self-efficacy to prevent acquiring SARS-CoV-2. Lower educational levels and low socio-economic status were less likely to be associated with desirable behaviours such as mask wearing when using public transport and social distancing. The finding that lower educational levels were associated with lower levels of knowledge on both the cause and spread of COVID-19 could suggest that public messaging is not sufficiently tailored to low literacy groups. Similarly, behaviours to prevent exposure to COVID-19 were practised less by those with lower education and socio-economic status. While lower levels of knowledge about these behaviours could account for this, lack of social distancing could be related to a lack of space for those from more crowded areas such as informal settlements. For some of the statistically significant findings, the magnitude of the difference was relatively small and may not be practically relevant. For example, although younger age was associated with greater knowledge on spread of COVID-19, knowledge levels were high for all three age groups and ranged from 92.8% to 95.5%. The finding that older age was the only significant sociodemographic predictor for intention to get vaccinated likely reflects the perceived and actual vulnerability of this age group. While several desired preventive behaviours led to a greater likelihood of intending to vaccinate, wearing a face mask due to COVID-19 was the only behaviour that remained significant after controlling for socio-demographic variables and confounders. Mask wearing has been the most common preventive practice and given the inconvenience, cost and social barrier experienced by wearing a mask, vaccination prior to its availability as a desired alternative is plausible. Since few studies have explored KAP related to COVID-19 prevention in South Africa, these findings, based on a household survey with a large sample size, add to the body of knowledge on COVID-19 prevention in South Africa. In a scoping review of studies that investigated KAP in Africa, none of the 28 studies that fulfilled the inclusion criteria, which included articles published between December 2019 and October 2020, were from Southern Africa [8]. Previous COVID-19 KAP studies in South Africa have focused on specific occupation groups and/or have used telephonic interviews. For example, the NIDS-CRAM study, a nationally representative survey that investigated vaccine acceptance, used computer assisted telephone interviews to collect data. [9]. Notwithstanding these different methodological approaches, earlier findings are similar to those documented in this study. In 2021 Moodley *et al*. conducted a survey of 286 health care workers in South Africa and found that high knowledge levels were associated with occupational category (doctor or nurse), who tend to represent a higher socio-economic status and educational level [10]. The NIDS CRAM study conducted in 2020 found that of the 91% of respondents who reported changing their behaviour in response to COVID-19 found that mask wearing was more common than social distancing (53% and 25% respectively) even though the proportions were lower than in the current study [11]. Further analysis of these data by Kollamparambil and Oyenubi found that higher socio-economic status and education levels were predictors of preventive behaviour such as social distancing and mask wearing [12]. They argue that while awareness of preventive measures is high, barriers to adoption of such measures are influenced by socio-economic context.

Several KAP studies in other African countries have shown that high levels of COVID-19 knowledge were not necessarily associated with high levels of preventive practice with few studies showing that the wearing of face masks and hand hygiene were a priority [8]. This finding is not dissimilar to other areas of behaviour change, and to the results of this study-high levels of knowledge of COVID-19 spread were not translated to similarly high levels of regular handwashing nor social distancing. To shift these behaviours, carefully designed social and behaviour change programmes need to target those with lower levels of educational and socio-economic status, while taking account of structural barriers such as lack of access to running water and/or soap for handwashing. Given the finding that television and radio are the most trusted source of COVID-19 information, these media remain important vehicles for behaviour change messaging. While vaccine roll-out had not begun when this study was conducted, determining intention to be vaccinated was measured to define factors likely to be associated with vaccine uptake. The Theory of Planned Behaviour suggests that intention to act is an important step in actually doing so [13]. Determining intention to get vaccinated, as well as the predictors and reasons for such intentions will help in designing messaging for vaccination communication campaigns both for COVID-19 and other respiratory illnesses [14]. In this study, 64.7% of the population intended to vaccinate and this was significantly associated with being 65 years or older and increased mask wearing. Using similar categories to measure vaccine intention (strongly agree and somewhat agree), the NIDS-CRAM study found a high proportion, 71%, would have a vaccine if it were available. Since a national sample using telephone-based interviews was used in the latter study, differences in socio-demographic factors of the sample could account for this difference. As of 21 July 2022, vaccine coverage has indeed been higher in older age groups-with 69.5% of females and 73.4% of males older than 60 years vaccinated compared to 42.8% and 33.0% respectively in the 18-34 year age group [15]. Actual vaccination in the entire population was lower than those who intended to vaccinate-43.7% of males and 56.2% of females were vaccinated in July 2022 compared to 64.7% who intended to. In South Africa, a recent review of seven surveys found that willingness to receive COVID-19 vaccination ranged from 52%-82% [16]. Three of these studies assessed reasons for vaccine hesitancy and all found concerns about vaccine side effects to be the most common reason. This study concurred with these findings suggesting that public information about the safety, side effects and benefits of the vaccine will help to promote vaccine uptake. The finding that older people had higher odds of intending to get vaccinated could reflect their greater perceived and actual risk for disease and death. This finding is consistent with similar studies elsewhere [16]. Increasing demand and closing the intention-action gap in this target group needs to be prioritized. It was beyond the scope of this study to follow-up with participants and compare intention versus actual vaccination. Despite this, in South Africa, numbers vaccinated in the > 64 year age group have been lower than anticipated (even though higher than other groups) with around 3.9 million of the over 5 million in the target group vaccinated [15]. Since this represents a large number of vulnerable and unvaccinated people, household registration support through community healthcare workers, as well as food and/or travel vouchers incentives could be considered. Another strategy to close the intention action gap, in all age groups, is the use of intention prompts, such as writing down the date one plans to get vaccinated. These have been shown to be effective in increasing influenza vaccine rates [17]. Similarly, short message service (SMS) reminders, especially those using the text “reserved for you” and “is waiting for you” have been demonstrated to reduce the intention-action gap [18,19]. Importantly, reducing barriers such as logistics is another strategy to capitalize on positive intention.

**Limitations:** limitations of this study include social desirability bias where participants may have overstated their preventive behaviors/intention to get vaccinated and lack of measurement of vaccine knowledge which is a potential predictor of intention to get vaccinated. Since the study was conducted in three densely populated communities in South Africa, the findings do not represent the country as a whole. The results are likely to be different in rural areas, where access to infrastructure (eg. water for handwashing), masks, information and vaccines may be more challenging. The study did not consider the role of the respondent and/or a relative having previously contracted COVID-19 and this could have influenced their attitude to vaccination. For variables with missing data we retained missing as a category in the multivariable analysis, in some circumstances these were statistically significant in the final model. These results are difficult to interpret but may suggest that non-responders differ from individuals who did respond to the questions in unmeasured ways potentially associated with the outcome of interest.

## Conclusion

This study confirms the findings of previous research that has demonstrated the importance of socio-economic and educational status in preventive responses to COVID-19. Moreover, it finds that older age is an important predictor of intention to seek vaccination and highlights approaches to address the intention-action gap. Future research that follows up participants who intended to be vaccinated with actual vaccination using mixed methods could assist in understanding shifts from initial intention, highlighting the limitations in KAP studies and helping to measure the intention action gap.

### What is known about this topic

- *Knowledge, attitudes and practices data for COVID-19 is well researched in several other countries; the approaches used in these surveys were applied in our study*.

### What this study adds

- *This study provides knowledge, attitudes and practices data related to COVID-19 prevention in South Africa based on a household survey with a large sample size;*
- *It confirms the importance of considering socio-demographic factors when designing interventions to promote preventive behaviours for COVID-19 and other future respiratory epidemics*.
